# CuBi_2_O_4_ Synthesis, Characterization, and Application in Sensitive Amperometric/Voltammetric Detection of Amoxicillin in Aqueous Solutions

**DOI:** 10.3390/nano11030740

**Published:** 2021-03-15

**Authors:** Raluca Dumitru (m.Vodă), Sorina Negrea, Cornelia Păcurariu, Adrian Surdu, Adelina Ianculescu, Aniela Pop, Florica Manea

**Affiliations:** 1 Faculty of Industrial Chemistry and Environmental Engineering, Politehnica University Timisoara, Piata Victoriei No. 2, 300006 Timisoara, Romania; raluca.voda@upt.ro (R.D.(m.V.)); cornelia.pacurariu@upt.ro (C.P.); aniela.pop@upt.ro (A.P.); 2 National Institute of Research and Development for Industrial Ecology (INCD ECOIND), 300431 Timisoara, Romania; negrea.sorina@yahoo.com; 3 Department of Environmental Engineering and Management, “Gheorghe Asachi” Technical University of Iasi, 700050 Iasi, Romania; 4 Department of Oxide Materials Science and Engineering, Faculty of Applied Chemistry and Materials Science, Polytehnic University of Bucharest, Gh. Polizu Street No. 1–7, 011061 Bucharest, Romania; adrian.surdu@live.com (A.S.); a_ianculescu@yahoo.com (A.I.)

**Keywords:** CuBi_2_O_4_ electrocatalyst, amoxicillin, electrochemical detection, CuBi_2_O_4_-carbon nanofiber paste electrode

## Abstract

CuBi_2_O_4_ synthesized by thermolysis of a new Bi(III)-Cu(II) oxalate coordination compound, namely Bi_2_Cu(C_2_O_4_)_4_·0.25H_2_O, was tested through its integration within carbon nanofiber paste electrode, namely CuBi/carbon nanofiber (CNF), for the electrochemical detection of amoxicillin (AMX) in the aqueous solution. Thermal analysis and IR spectroscopy were used to characterize a CuBi_2_O_4_ precursor to optimize the synthesis conditions. The copper bismuth oxide obtained after a heating treatment of the precursor at 700 °C/1 h was investigated by an X-ray diffraction and scanning electron microscopy. The electrochemical behavior of CuBi/CNF in comparison with CNF paste electrode showed the electrocatalytic activity of CuBi_2_O_4_ toward amoxicillin detection. Two potential detections, with one at the potential value of +0.540 V/saturated calomel electrode (SCE) and the other at the potential value of −1.000 V/SCE, were identified by cyclic voltammetry, which were exploited to develop the enhanced voltammetric and/or amperometric detection protocols. Better electroanalytical performance for AMX detection was achieved for CuBi/CNF using differential-pulsed and square-wave voltammetries than others reported in the literature. Very nice results obtained through anodic and cathodic currents recorded at +0.750 V/SCE and −1.000 V/SCE in the same time period using a pseudo multiple-pulsed amperometry technique showed the great potential of the CuBi/CNF paste electrode for practical applications in amoxicillin detection in aqueous solutions.

## 1. Introduction

Currently, the sensing field is continuously evolving to reach demand in various practical applications, e.g., medical, pharmaceuticals, food, and environmental quality. Electrochemical sensors represent the main category of sensors due to their advantages of fast response, simplicity, and versatility. However, their electroanalytical performance depends on the electrode type correlated with the electrochemical techniques. Carbon-based electrodes are intensively used in the electroanalysis, but the main disadvantage is given by the slow rate of the electron transfer at the carbon surface that confers low sensitivity and implicit, limited applications. Nanostructured carbon offers a partial solution for the mentioned problem due to its enhanced electrocatalytic activity depending on the carbon type, but its high price restricts the practical applications. The carbon nanofiber is one of the cheapest categories of nanostructured carbon, which is vastly studied in electroanalysis both as a working electrode [1,2] and as the substrate for further modification to enhance its sensing characteristics [3,4,5]. Copper bismuth oxide (CuBi_2_O_4_) is receiving growing attention as a promising material for application in photocatalysis [6,7], photoelectrochemical water splitting [8,9], and sensing [10,11,12,13]. The properties of CuBi_2_O_4_ are in relation to the synthesis method. To date, many methods have been developed for preparing CuBi_2_O_4_ as coprecipitation [14], solvothermal [15] and hydrothermal methods [16,17,18], combustion [19], sono-chemical reactions [20], etc.

In this study, the electrocatalytic properties of copper bismuthate was studied using carbon nanofiber (CNF) as a substrate and for a comparison in detecting amoxicillin in the aqueous solution. Amoxicillin (AMX) belongs to the third generation of antibiotic-penicillin class and very frequently prescribed against a wide range of infections. It is also used in human and veterinary medicine. In this context, its presence in food (e.g., milk, eggs, meat) has been reported [21] and, also, it was detected in water environments in drinking water via hospital effluents and municipal wastewater as main sources [22]. AMX belongs to the emerging pollutants class from antibiotics-based pharmaceuticals being one of the eight substances listed in the “watch list” of substances for union-wide monitoring in the field of a water policy pursuant to Directive 2008/105/EC of the European Parliament and of the Council and repealing Commission Implementing Decision (EU) 2015/495 [23]. As other pharmaceuticals, its quantitative and qualitative detection methods are urgently required and several methods including chromatography, spectroscopy, and spectro-fluorometry have been used to determine amoxicillin [24,25,26]. Due to drawbacks related to cost, time consumption, a separate stage of sample preparation, and research for the development of a fast and easy determination method of AMX is required. The electrochemical methods should be regarded as a viable alternative for AMX determination by taking into account their advantages of a fast and simple detection method. Several electrochemical detection methods of AMX using carbon-based and modified electrodes have been reported [26,27,28], but more improvement of the electroanalytical performance is required for which new composition of the electrode, which represents the core of the electrochemical detection, should be developed and tested. Copper bismuth oxide synthesized by thermolysis of Bi(III)-Cu(II) oxalate coordination compound at 700 °C/h was used to modify the carbon nanofiber paste by simple mixing in paraffin oil as a CuBi/CNF electrode to enhance the performance of the electrochemical detection of amoxicillin. The new electrode was tested comparatively with CNF paste electrode in AMX detection using conventional and advanced voltammetric and amperometric techniques.

## 2. Materials and Methods

### 2.1. Synthesis and Characterization of CuBi_2_O_4_

For the synthesis of the oxalate coordination compound, Bi(NO_3_)_3_·5H_2_O, Cu(NO_3_)_2_·3H_2_O, 1,2-ethanediol, and 2 M nitric acid solution were used as reagents from Merck (Darmstadt, Germany). Copper bismuth oxide was obtained through thermal decomposition of the precursor in the temperature range of 500–700 °C. A water solution containing bismuth nitrate, copper nitrate, 1,2-ethanediol, and nitric acid (2 M) in a molar ratio 2:1:4:2.66 was used. This mixture was heated in a water bath for about 30 min. The reaction is finished when no more gas is evolved. The resulting solid product was purified by washing with acetone and dried under a room temperature environment. The coordination compound Bi_2_Cu(C_2_O_4_)_4_·0.25H_2_O is synthesized using 2 M nitric acid solution.

In order to obtain copper bismuth oxide powders, the oxalate coordination compound was thermally treated in air, for 1 h, with a heating rate of 10 °C min^−1^ in the temperature range of 500–700 °C.

FTIR (Fourier-transform infrared) spectrum (KBr pellets) of the coordination compound was recorded on a Jasco FT-IR spectrophotometer (Jasco, Tokyo, Japan), in the range of 4000–400 cm^−1^. Thermal measurements through thermal gravimetry (TG), differential thermogravimetry (DTG) and differential scanning calorimetry (DSC) were performed on the precursor using a NETZSCH-STA 449C instrument (Netzsch Group, Selb, Germany) in the range of 25–700 °C, using alumina crucibles. The experiments were carried out in artificial air flow of 20 mL min^−1^ and a heating rate of 10 K min^−1^.

The phase purity and crystal structure of calcined powders were determined by using X-ray diffraction (XRD) analyses performed at room temperature by means of a Rigaku Ultima IV diffractometer (Rigaku Co., Tokyo, Japan), using Ni-filtered CuKα radiation (λ = 1.5418 Ǻ), with a scan step increment of 0.01° and a scanning rate of 1 °/min, for 2*θ* ranged between 20–80°.

The size and the agglomeration tendency of the CuBi_2_O_4_ particles was assessed by a scanning electron microscopy (FE-SEM), using a high resolution FEI QUANTA INSPECT F microscope with a field emission gun (FEI Co., Eindhoven, The Nederlands).

### 2.2. Copper Bismuthate-Carbon Nanofiber Paste Electrode (CuBi/CNF) Obtaining and Electrochemical Characterization

The copper bismuthate-carbon nanofiber paste electrode (CuBi/CNF) was obtained by mixing certain amounts of carbon nanofibers, paraffin oil, and copper bismuth to reach the ratio of 21.5 wt. % carbon nanofibers, 43 wt. % copper bismuth, and 35.5 wt. % paraffin oil. For comparison, a carbon nanofiber paste electrode (CNF) was similarly obtained with the composition of 64.5 wt. % carbon nanofibers and 35.5 wt. % paraffin oil. The mass ratio of copper bismuth, carbon nanofibers, and paraffin oil of 2:1:1.65 was chosen to assure the sufficient contribution of copper bismuthate, the electrode stability, and conductivity. For comparison, the ratio of 3:1.65 carbon nanofibers to paraffin oil as the carbon nanofiber paste was used. The carbon nanofibers (>98% purity) and paraffin oil were of an analytical standard, provided by Sigma Aldrich (Darmstadt, Germany).

Prior to each detection experiment, the electrode was electrochemically activated and stabilized by 9 cyclic voltammograms using a cyclic voltammetry (CV) technique within the potential range between −1.5 and +1.0 V/saturated calomel electrode (SCE in the 0.1 M Na_2_SO_4_ supporting electrolyte.

### 2.3. Electrochemical Detection of AMX

All the electrochemical experiments were performed using a classical three electrodes cell, having the saturated calomel electrode (SCE) as a reference electrode, platinum as a counter electrode, and the CuBi/CNF and respective CNF paste electrodes as the working electrode. The electrodes were connected to an Autolab potentiostat/galvanostat PGSTAT 302 (Eco Chemie, The Netherlands) controlled with GPES 4.9 software.

The experiments were performed in the 0.1 M Na_2_SO_4_ 0.1 M supporting electrolyte, and the applied techniques were cyclic voltammetry (CV), differential-pulsed voltammetry (DPV), and square-wave voltammetry (SWV) as voltammetric techniques as well as chronoamperometry (CA) and multiple-pulsed amperometry (MPA) as amperometric techniques.

The lowest limit of detection (*LOD*) and limit of quantification (*LOQ*) were calculated through the following equation, i.e., LOD = 3·SD·m^−1^ and LOQ = 10·SD·m^−1^, where *SD* is the standard deviation of 5 blanks and *m* is the slope of the analytical plots [29].

## 3. Results

### 3.1. Characterization of Bi_2_Cu(C_2_O_4_)_4_·0.25H_2_O Oxalate Precursor

The synthesis method of the oxalate coordination compound is based on the redox reaction between 1,2-ethanediol and nitrate ion:

(1) $$\begin{array}{c} 12{\;C}_{2}H_{4}{\left(\mathrm{OH}\right)}_{2}+\;6\;({\left[\mathrm{Bi}{\left({\mathrm{OH}}_{2}\right)}_{6}\right]}^{3+}+\;3\mathrm{NO})\;+\;3\;({\left[\mathrm{Cu}{\left({\mathrm{OH}}_{2}\right)}_{4}\right]}^{2+}{+\;2\mathrm{NO})\;+\;8\;(H}^{+}+\;\mathrm{NO})\overset{\Delta t{}^{\circ}}{\to }3\;\\ {\;\mathrm{Bi}}_{2}\mathrm{Cu}{\left(C_{2}O_{4}\right)}_{4}\cdot {0.25H}_{2}O_{\left(s\right)}{+\;32\mathrm{NO}}_{\left(g\right)}+{87.25H}_{2}O_{\left(g\right)} \end{array}$$

**Figure 1:**
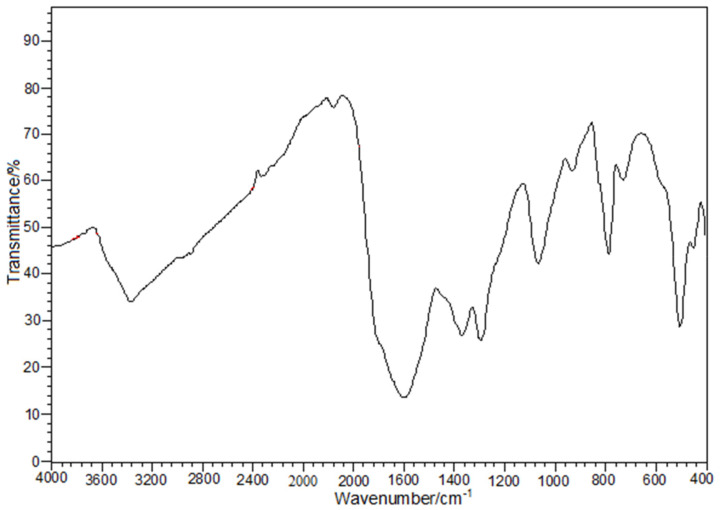
The IR vibrational spectrum of Bi_2_Cu(C_2_O_4_)_4_·0.25H_2_O compound.

The IR spectrum of the synthesized coordination compound is provided in Figure 1.

NO_(g)_ + ½ O_2(g)_ → NO_2(g)_ (2)

Besides the water presence [3371 cm^−1^ (ν_OH_, ν_H2O_), 700–800 cm^−1^ (lattice water)], two different coordination modes for oxalate anions as tetradentate bridges [1602 cm^−1^ (ν_asym OCO_), 1368 cm^−1^ (ν_sym OCO_) and 930 cm^−1^ (δ_OCO_)] and as chelate bidentate [1709 cm^−1^ (ν_asym OCO_), 1294 cm^−1^ (ν_sym OCO_)] were identified [30,31]. The band at 1064 cm^−1^ is assigned to the vibration ν_(C-O)_. The coordination of oxalate anion is confirmed by the bands lying in 500–400 cm^−1^ [ν_(Bi-O)_ and ν_(Cu-O)_ vibrations] [32,33].

**Figure 2:**
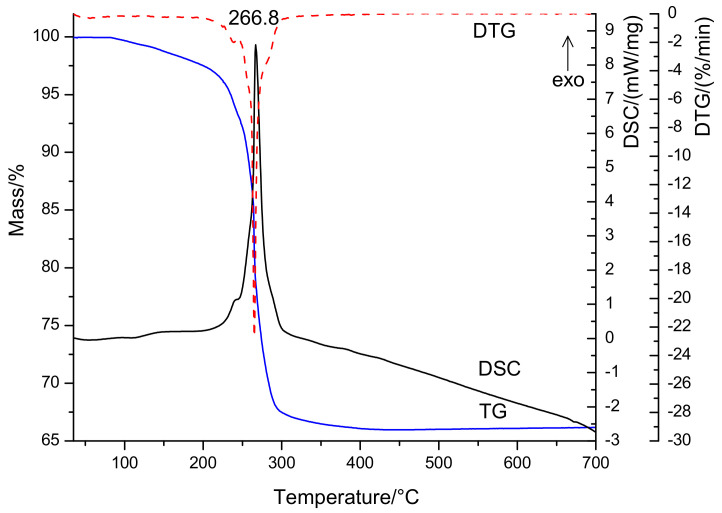
Thermal curves DSC, TG, and DTG of Bi_2_Cu(C_2_O_4_)_4_·0.25H_2_O compound.

The thermal decomposition of the investigated coordination compound occurs in the temperature range of 25–700 °C (Figure 2) and confirm the formation as the end decomposition product of a compound with a molecular formula of CuBi_2_O_4_ (mass loss calcd./found (%): 34.91/34.00). The first decomposition stage of the Bi_2_Cu(C_2_O_4_)_4_·0.25H_2_O compound associated with an endothermic effect is attributed to the dehydration reaction that implies the evolution of the lattice water molecules (25–150 °C, mass loss: found 0.41%; calcd. 0.54%). The second decomposition step (150–400 °C) associated with an exothermic effect is assigned to the degradation of the oxalate anions (mass loss, found 33.59%, calcd. 34.37%) with the formation of amorphous CuBi_2_O_4_.

The crystallized copper bismuth oxide was obtained starting with 500 °C. Based on the above presented results, this study proposed the following mechanism for the thermal decomposition of a bismuth-copper oxalate precursor.

(3) $$\left[{\mathrm{Bi}}_{2}\mathrm{Cu}{\left(C_{2}O_{4}\right)}_{4}\right]\cdot {0.25H}_{2}O_{\left(s\right)}\overset{\;(I)}{\to }\;{0.25H}_{2}O_{\left(g\right)}+\;{\left[{\mathrm{Bi}}_{2}\mathrm{Cu}{\left(C_{2}O_{4}\right)}_{4}\right]}_{\left(s\right)}$$

(4) $${\left[{\mathrm{Bi}}_{2}\mathrm{Cu}{\left(C_{2}O_{4}\right)}_{4}\right]}_{\left(s\right)}\overset{\;(II)}{\to }{\;4\;\mathrm{CO}}_{2(g)}{+\;4\;\mathrm{CO}}_{\left(g\right)}{+\;\mathrm{CuBi}}_{2}O_{4(s)}$$

### 3.2. Characterization of the CuBi_2_O_4_ Powder

**Figure 3:**
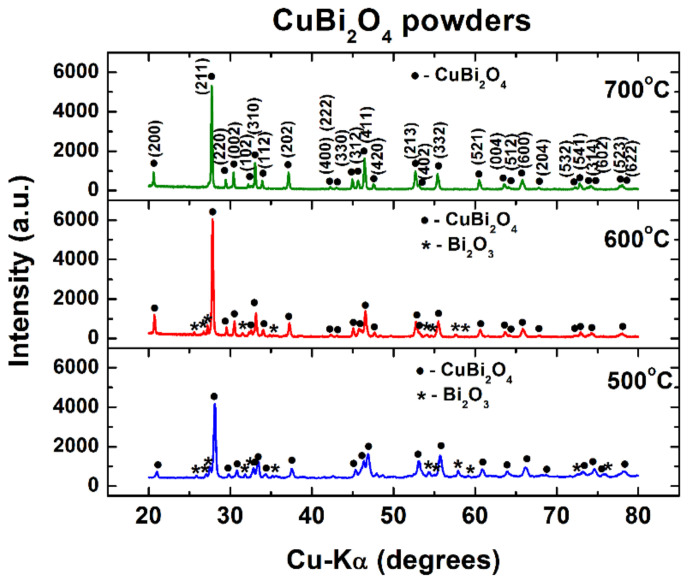
X-ray diffraction (XRD) patterns at room temperature for the CuBi_2_O_4_ powders calcined at various temperatures.

The X-ray diffraction patterns prove that the crystallization process starts at 500 °C, when CuBi_2_O_4_ with a tetragonal structure was identified as a major phase by its main diffraction lines. Small amounts of Bi_2_O_3_ was also detected as a secondary phase in the powders calcined at 500 and 600 °C, respectively (Figure 3). A heating treatment of the coordination compound as a precursor performed at 700 °C for one hour determines the formation of pure CuBi_2_O_4_, as shown by the XRD pattern (Figure 3). The structural parameters of the powders under investigation obtained by Rietveld refinement are summarized in Table 1. As expected, the increase of the calcination temperature induces the increase of the average crystallite size and, consequently, a decrease of the internal strains. One can observe that the increase of the crystallite size involves a decreasing evolution of the “in- plane” *a* and *b* parameters of the tetragonal unit cell of CuBi_2_O_4_, while the variation of the out-of-plane parameter *c* is non-monotonic.

**Figure 4:**
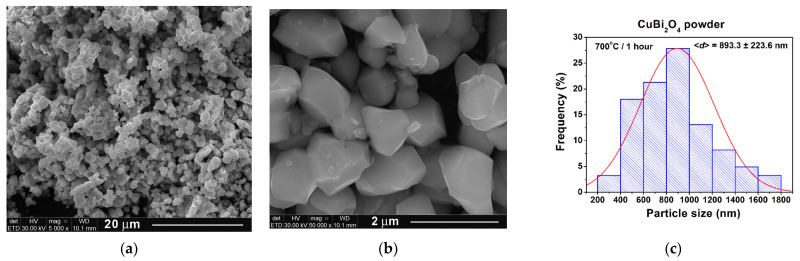
FE-SEM images the CuBi_2_O_4_ powder calcined at 700 °C for 1 h: (**a**) low magnification (×5000) overall view, (**b**) high magnification view (×50,000), and (**c**) histogram indicating the particle size distribution.

FE-SEM investigations were performed only on the single phase CuBi_2_O_4_ powder calcined at 700 °C for 1 h. The low magnification image of Figure 4a shows the presence of irregular particles, which exhibit a clear tendency to form aggregates due to a presintering process induced by the higher calcination temperature [34]. The high magnification image of Figure 4b reveals that most of the particles exhibit a polyhedral shape with well-defined faces, edges, and corners and various sizes that ranged between 200 and 1800 nm. An average particle size of 893.3 nm was estimated based on the histogram of the particle size distribution of Figure 4c. Taking into account the value of the average crystallite size determined from the XRD data, one can assume that polycrystalline particles, consisting of a variable number of crystallites, were obtained after calcination at 700 °C for 1 h.

### 3.3. Application in Electrochemical Sensing of Amoxicillin (AMX)

The electrocatalytic activity of CuBi_2_O_4_ was tested by modifying a carbon nanofiber paste electrode as CuBi/CNF to enhance the electrocatalytic detection of amoxicillin (AMX).

**Figure 5:**
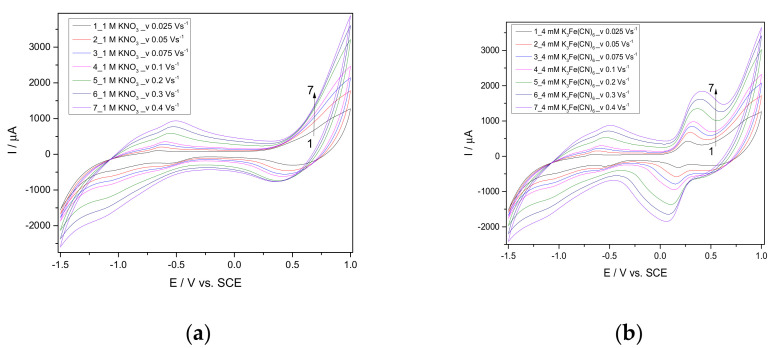
Cyclic voltammograms (CV) recorded at a CuBi/CNF modified electrode in a 1 M KNO_3_ supporting electrolyte (**a**) and the presence of 4 mM K_3_[Fe(CN)_6_] (**b**) at different scan rates: 0.025, 0.05, 0.075, 0.1, 0.2, 0.3, and 0.4 Vs^−1^.

Before detection testing, the CuBi/CNF electrode was characterized electrochemically by cyclic voltammetry (CV) using classical potassium ferri/ferrocyanide redox system to determine its electroactive area. Cyclic voltammograms (CV) of 4 mM K_3_[Fe(CN)_6_] in a 1 M KNO_3_ supporting electrolyte were recorded at different scanning rates. The results are presented in Figure 5a,b.

The diffusion coefficient was determined comparatively for both CuBi/CNF and CNF paste electrodes according to the Randles–Sevcik Equation (5):

(5) $$I_{p}=2.69\times 10^{5}AD^{1/2}n^{3/2}v^{1/2}C$$

where *A* represents the area of the electrode (cm^2^), *n* is the number of electrons participating in the reaction (and is equal to 1), *D* is the diffusion coefficient of the molecule in the solution, *C* is the concentration of the probe molecule in the solution and is 4 mM, and *v* is the scan rate (V s^−1^). The linear dependence between the current peak and the square root of the scan rate allowed determining the diffusion coefficient of 2.70×10^−5^ cm^2^ s^−1^ for the CuBi/CNF electrode and respective 7.86 × 10^−6^ cm^2^ s^−1^ for the CNF electrode. Taking into account the theoretical diffusion coefficient value of 6.70 × 10^−6^ cm^2^ s^−1^ found in the literature data [35], the value of the electroactive electrode area was determined to be 0.790 cm^2^ for the CuBi/CNF electrode of 0.230 cm^2^ for the CNF electrode vs. a 0.196-cm^2^ geometric area value of the electrode. It is clear that CuBi/CNF exhibited a much higher electroactive area in comparison with one of the CNF electrodes.

#### 3.3.1. Cyclic Voltammetry

Cyclic voltammetry (CV) was applied to characterize the electrochemical behavior of CuBi/CNF electrode in comparison with a CNF paste electrode in a 0.1-M Na_2_SO_4_ supporting electrolyte and in the presence of different AMX concentrations.

**Figure 6:**
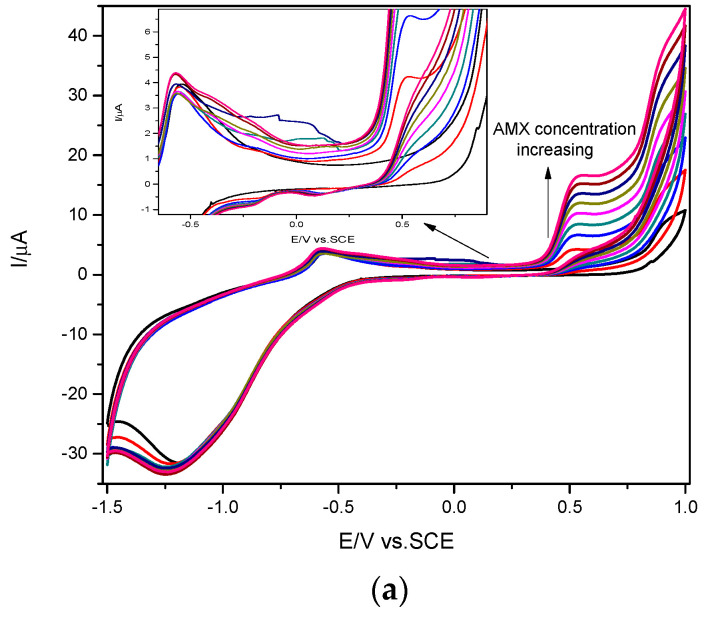

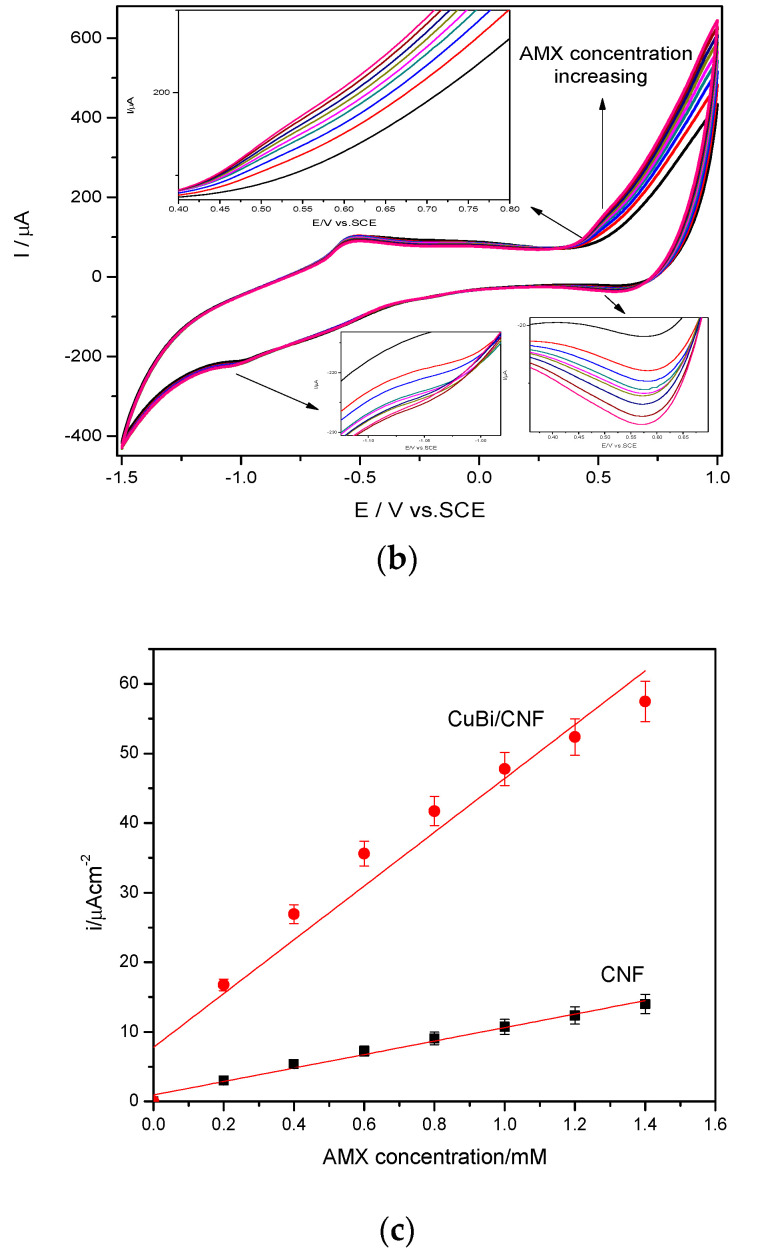
Cyclic voltammograms recorded at the 0.5 Vs^−1^ scan rate on the CNF paste electrode (**a**) and CuBi/CNF modified paste electrode (**b**) in 0.1 M Na_2_SO_4_ supporting electrolyte and in the presence of various AMX concentrations ranged from 0.2 mM to 1.6 mM AMX concentration. Comparative calibration plots for AMX detection in the concentration range from 0.2 to 1.4 mM, rerecorded at the potential value of +0.55 V/SCE (**c**).

The presence of CuBi_2_O_4_ on the electrode surface determined a significant increase of the background current due to its capacitive behavior related to the morphostructural properties and the electro active surface area. It can be noticed that the oxidation of AMX started at the potential value of +0.5 V/SCE for both electrodes and the anodic peak current increased linearly with AMX concentration (see Figure 6). The linearity between the anodic current recorded at the potential value of +0.54 V/SCE and the AMX concentration allowed us to determine the detection sensitivity, which is higher for modified CuBi/CNF (181 µA mM^−1^ cm^−2^) in comparison with the CNF paste electrode (133 µA mM^−1^ cm^−2^) (the results of linearizations are not shown here). The electrochemical response is based on the oxidation peak, which characterizes the one electron involving an oxidation reaction of the phenolic substituent to a respective carbonyl group on the side chain of the AMX molecule [28]. One corresponding cathodic peak due to the reduction process is noticed for CuBi/CNF in comparison with the CNF electrode that did not exhibit the cathodic peak in this anodic range. This aspect suggests that the cathodic peak corresponded to the CuBi_2_O_4_ presence. According to the literature [10], in the anodic potential range, Cu^3+^ species generated during scanning in the CuBi_2_O_4_ matrix are involved in the AMX oxidation and, by reverse scanning, the reduction of Cu^3+^ to Cu^2+^ species can be noticed. Thus, a quasi-reversible redox couple at the potential value of about +0.54 V/SCE resulted from the intrinsic redox properties of CuBi_2_O_4_. However, a reduction peak that increased with AMX concentration rising appeared at the potential value of about −1.0 V/SCE, which was noticed for the CuBi/CNF electrode (Figure 5). According to the literature [14], the reduction peak corresponds to the BiO_2_^−^ reducing to BiO_2_^2−^, which is involved in the reduction process of AMX or the AMX oxidation product.

#### 3.3.2. Influence of the Scan Rate

**Figure 7:**
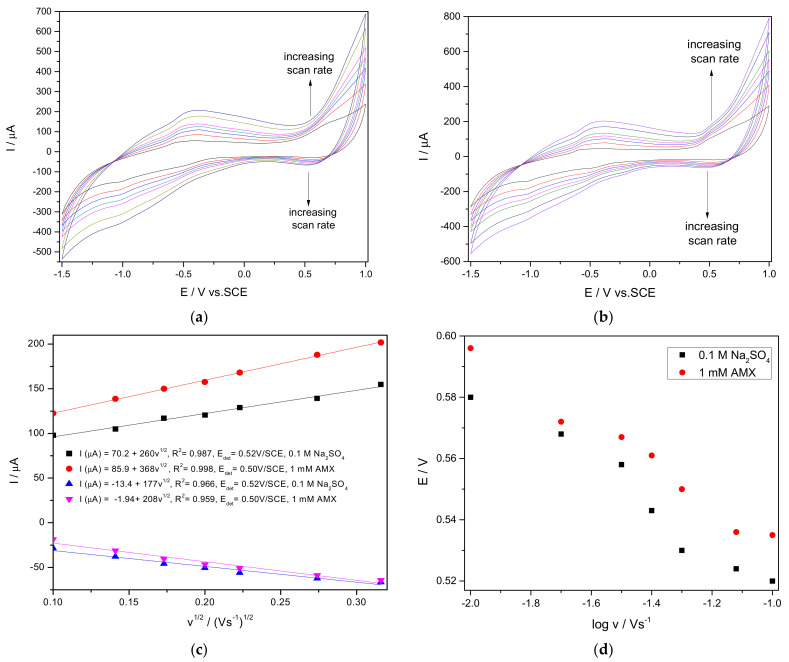
Cyclic voltammograms recorded at CuBi/CNF modified paste electrode in 0.1 M Na_2_SO_4_ supporting electrolyte (**a**) and 1 mM AMX (**b**) at various scan rates: (1) 10, (2) 20, (3) 30, (4) 40, (5) 50, (6) 75, and (7) 100 mVs^−1^. Dependence of anodic peak current vs. square root of scan rate (**c**). Dependence of anodic and cathodic peak potentials vs. logarithm of the scan rate (**d**).

To elucidate some mechanistic aspects, the influence of the scan rate on the electrochemical behaviour of CuBi/CNF in the absence/presence of 1 mM AMX and the results are presented in Figure 7.

The electrochemical behaviour of CuBi/CNF in the absence and in the presence of AMX is similar to the shape. The anodic peak current recorded at +0.54 V/SCE increased linearly with the scan rate increasing. After the addition of 1 mM AMX, the anodic peak current significantly increased, while the corresponding cathodic peak decreased (see Figure 7c), which indicates that the AMX oxidation involves an electrochemical reaction coupled with a chemical reaction, which is an electrocatalytic reaction. No major changes in anodic peak potential with the scan rate increasing are noticed, which are slightly shifted to more negative values, indicating the redox system reversibility in both the absence and the presence of AMX (Figure 7d). It can be concluded that the active species responsible for the redox couple recorded at about +0.54 V/SCE plays an important role in electro-catalyzing the oxidation of AMX.

To enhance the electroanalytical performance for AMX detection, the electrochemical behaviour of the electrode in the presence of AMX and the operating characteristics of the voltametric and amperometric techniques are further considered.

#### 3.3.3. Differential-Pulsed Voltammetry (DPV) and Square-Wave Voltammetry (SWV)

**Figure 8:**
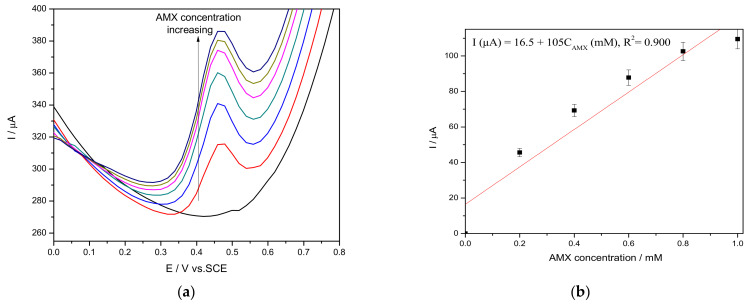
(**a**) Differential-pulse voltammograms recorded on the CuBi/CNF electrode in 0.1 M Na_2_SO_4_ supporting electrolyte in the presence of various AMX concentrations: 0.2 mM, 0.4 mM, 0.6 mM, 0.8 mM, 1.0 mM, and 1.2 mM. SP of 0.02 V and MA of 0.10 V. (**b**) Calibration plots for AMX detection in the concentration range of 0.2–1.2 mM recorded at the potential value of E = +0.500 V/SCE.

In comparison with CV, the electrochemical response provided by the differential-pulsed voltammetry technique is superior regarding the sensitivity, accuracy, and resolution for electrochemical sensing and electrochemical mechanism elucidation [36,37]. In AMX detection, DPV was tested under various operating conditions related to the step potential (SP), which ranged from 0.01 to 0.05 V while modulation amplitude (MA) ranged from 0.05 to 0.2 V. The shapes of DPVs are different when related to the operating conditions. The electroanalytical parameters are influenced by the electrochemical response stability. The best electroanalytical results were achieved for SP of 0.02 V and MA of 0.1 V corresponding to a 20-mVs^−1^ scan rate (Figure 8).

**Figure 9:**
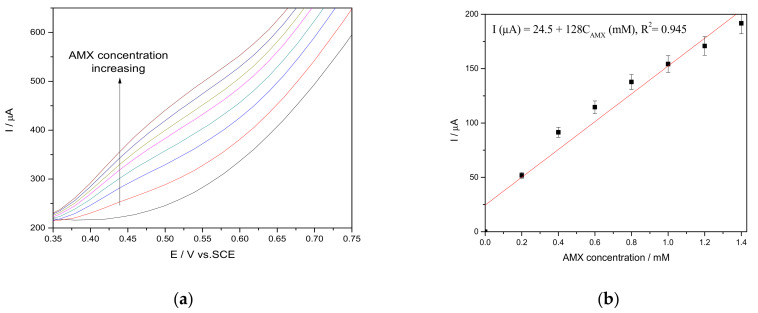
(**a**) Square-wave voltammograms recorded on CuBi/CNF electrode in 0.1 M Na_2_SO_4_ supporting electrolyte, in the presence of various AMX concentrations: 0.2 mM, 0.4 mM, 0.6 mM, 0.8 mM, 1.0 mM, 1.2 mM, and 1.4 mM. Step potential (SP) of 0.02 V, modulation amplitude (MA) of 0.1 V. f = 20 Hz. (**b**) Calibration plots for AMX detection in the concentration range 0.2–1.4 mM recorded at the potential value of E = +0.500 V/SCE.

It can be noticed that the detection potential value is slightly shifted to a lower potential value in comparison with CV, which is one of the main characteristics of the differential voltammetry and is an advantage of this technique as related to the detection aspect. Under these operating conditions, the best sensitivity for AMX detection was reached at the potential value of +0.500 V/SCE (538 µA mM^−1^ cm^−2^), which is much higher in comparison with one reached by CV (181 µA mM^−1^ cm^−2^). Furthermore, the square-wave voltammetry (SWV) technique was tested for a comparison with DPV under the previously mentioned, optimized, operating DPV conditions. Taking into account the major advantage of SWV given by speed controlled by the frequency product (f) and step potential (SP), the frequency ranged from 10 to 50 Hz. The best results were achieved for the frequency of 20 Hz at the scan rate of 0.4 V∙s^−1^ and the results are presented in Figure 9, which showed a stable and fast voltammetric response. All electroanalytical parameters reached for the voltametric techniques are shown comparatively in Table 2, and it can be noticed that DPV allowed reaching the lowest limit of detection of 1.5 10^−7^ M, while the best sensitivity (653 µA mM^−1^ cm^−2^) was achieved by SWV.

#### 3.3.4. Amperometry for AMX Detection on the CuBi/CNF Electrode

**Figure 10:**
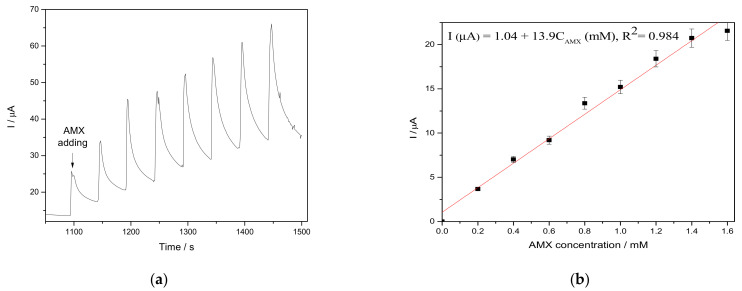
(**a**) Chronoamperograms recorded on CuBi/CNF electrode in 0.1 M Na_2_SO_4_ supporting electrolyte and in the presence of various AMX concentrations: 0.2 mM, 0.4 mM, 0.6 mM, 0.8 mM, 1.0 mM, 1.2 mM, and 1.4 mM at (**a**) an applied potential level of +0.750 V/SCE. (**b**) Calibration plots for AMX detection in the concentration range of 0.2 to 1.4 mM.

The amperometric techniques, considered as the simplest for practical applications, were tested in order to elaborate the enhanced amperometric detection protocol. This technique operates at one or more certain potential levels and the main disadvantage of chronoamperometry is the fast electrode fouling that means the loss of the amperometric signal. This aspect can be easily shown in sensitivity decreasing in comparison with CV results. Chronoamperograms recorded in the presence of the same AMX concentrations range (presented in Figure 10a) operated at one potential level of +0.750 V/SCE showed a sensitivity by about two times lower (Figure 10b) than the sensitivity obtained by CV due to possible electrode fouling.

**Figure 11:**
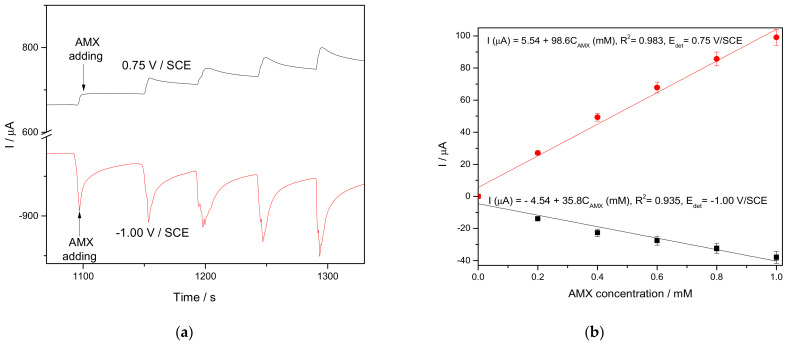
(**a**) Multiple-pulsed amperograms recorded at CuBi/CNF paste electrode in 0.1 M Na_2_SO_4_ supporting electrolyte in the presence of various AMX concentrations: 0.2 mM, 0.4 mM, 0.6 mM, 0.8 mM, and 1.0 mM, at two applied potential levels, i.e., +0.750 V/SCE and −1.00 V/SCE. (**b**)Calibration plots for AMX detection in the concentration range of 0.2–1.0 mM recorded at the potential +0.750 V/SCE and −1.00 V/SCE.

In order to enhance the electroanalytical performance of amperometric detection of AMX, the pseudo multiple-pulsed amperometry (MPA) technique, operated at the two potential values corresponding to the reduction and the oxidation processes, was tested and the results of the amperograms and corresponding calibration plots are gathered in Figure 11a,b.

In general, it is well-known that the MPA technique exhibit the advantage of fouling avoiding for amperometric detection and is based on in-situ electrochemical activation of the electrode surface through fast and short amperometric pulses [38]. In this application, the MPA technique is adapted and named pseudo-MPA due to the electrode activation assured during the detection step without other supplementary pulses at the cathodic/anodic potential values, which are imposed for MPA. The anodic pulse level was selected at a potential value of +0.750 V/SCE, which is higher than the detection potential determined by CV and the cathodic one at the potential value of −1.00 V/SCE, in accordance with the CV results. The amperometric results are much better than voltammetric ones related to the sensitivity and it must be highlighted that the CuBi/CNF electrode potential for cathodic detection of AMX is a very promising aspect for simultaneous detection. The electroanalytical performance of detection achieved by the amperometry technique at the potential value of +0.750 V/SCE is comparable with one reached by the voltammetric techniques, which recommend this electrode and technique for further development of the amperometric-based protocol for AMX detection.

All detection results are presented in Table 2, and it can be noticed that CuBi/CNF is very promising for the AMX detection in aqueous solution.

The electroanalytical results related to the lowest limit of detection and the sensitivity obtained with the CuBi/CNF electrode are better than those reported by Essousi et al. [28], and showed the possibility of a practical application of this electrode in the detection of amoxicillin in the aqueous solution. The practical analytical application of the CuBi/CNF paste electrode using the DPV method was established by determining AMX in a water sample. Analyzing three parallel tap water samples spiked with 35 and 70 mg∙L^−1^ AMX were selected for the recovery test. Good recovery and reproducibility of the results were found based on the minimum recovery values of 95% and the maximum relative standard deviation (RSD) values of 6% for both concentrations.

## 4. Conclusions

In this paper, CuBi_2_O_4_ powders were prepared by a new method based on the thermolysis of the oxalate coordination compound starting with 500 °C. XRD and SEM analyses indicated that phase-pure CuBi_2_O_4_ particles with an average particle size of 893.3 nm were obtained after calcination at 700 °C for 1 h.

The electrocatalytic effect of CuBi_2_O_4_ particles toward the amoxicillin detection was tested using a carbon nanofiber electrode as a substrate through the CuBi_2_O_4_/CNF paste electrode (CuBi/BDD) and in comparison with the CNF paste electrode. Cyclic voltammetric studies showed the superiority of CuBi/CNF paste through the redox system manifested within intrinsic CuBi_2_O_4_.

The voltammetric detection methods based on differential-pulsed voltammetry operated under 0.02 V as a step potential and 0.100 V as a modulation amplitude that allowed reaching the lowest limit of detection of 0.15 µM, while a fast and stable response characterized by the highest sensitivity of 653 µA µM cm^−2^ was achieved with square-wave voltammetry operated under a 0.02 Vas step potential and a 0.100 V as a modulation amplitude, frequency of 20 Hz, and a scan rate of 0.4 V s^−1^. The amperometric detection method involving a pseudo multi-pulsed amperometry technique based on both anodic and cathodic potential levels led to very promising results for AMX detection related to sensitivity and selectivity. These results confirm the great potential of the CuBi/CNF paste electrode to be used for amoxicillin detection in the aqueous solution through the electrooxidation and electroreduction process, which should be further exploited for selective/simultaneous detection of amoxicillin in a multi-component matrix, considering a prior concentration stage that can be separate or included within the detection protocol by simple sorption onto the electrode surface.

## Figures and Tables

**Table 1:** Phase composition and structural parameters of CuBi_2_O_4_ powders prepared by the oxalate route and thermally treated for 1 h at various temperatures.

Calcination Temperature (°C)		500	600	700
Phase composition		CuBi_2_O_4_ (ICDD no. 01-080-1906)—61%, Bi_2_O_3_-m (ICDD no. 04-007-1342)—27.8%, Bi_2_O_3_-t (ICDD no. 01-073-6885)—11.2%.	CuBi_2_O_4_ (ICDD no. 01-080-1906)—83.2%, Bi_2_O_3_-m (ICDD no. 04-007-1342)—6.9%, Bi_2_O_3_-t (ICDD no. 01-073-6885)—9.9%.	CuBi_2_O_4_ (ICDD no.01-080-1906)—100%.
CuBi_2_O_4_ structure		Tetragonal, P4/ncc	Tetragonal, P4/ncc	Tetragonal, P4/ncc
Unit cell parameters	a (Å)	8.501143 ± 0.000912	8.500031 ± 0.000566	8.496553 ± 0.000555
	b (Å)	8.501143 ± 0.000912	8.500031 ± 0.000566	8.496553 ± 0.000555
	c (Å)	5.817769 ± 0.000817	5.827083 ± 0.000496	5.822091 ± 0.000478
	α = β = γ (°)	90	90	90
Unit cell volume, V (Å^3^)		420.4469	421.0097	420.3050
Expected *R,* R_exp_		11.26198	10.68391	10.97338
R profile, R_p_		9.08664	6.68487	7.20859
Weighted R profile, R_wp_		13.50014	9.18731	9.57705
Goodness of fit, χ^2^		1.43697	0.73946	0.7617
Crystallite size, <D> (nm)		29.79 ± 8.24	33.64 ± 5.65	54.00 ± 4.87
Internal strains, <S> (%)		0.29 ± 0.04	0.26 ± 0.06	0.17 ± 0.08

**Table 2:** The electroanalytical parameters for AMX electrochemical detection using the CuBi/CNF electrode.

Technique	Working Parameters	Detection Potential (V/SCE)	Sensitivity (µA µM^−1^ cm^−2^)	LOD (µM)	LOQ (µM)	R^2^
CV	v = 0.05 V s^−1^	+0.550	181	0.965	3.22	0.938
		−1.00	78.5	1.31	4.80	0.920
DPV	SP = 0.02 V	+0.500	538	0.150	0.520	0.946
	MA = 0.10 V					
	V = 0.20 V s^−1^					
SWV	SP = 0.02 V	+0.500	653	1.60	5.33	0.945
	MA = 0.10 V					
	f = 20 Hz; v = 0.20 V s^−1^					
CA		+0.750	70.9	5.87	19.6	0.984
MPA	two potential levels, pulse time = 0.10 s	+0.750	503	2.43	8.12	0.983
		−1.00	183	4.74	15.8	0.937
